# Relationship-Building Skills of Child-Rearing Mothers in Japanese Communities

**DOI:** 10.1155/2016/9091039

**Published:** 2016-06-26

**Authors:** Hikaru Honda, Nobuko Matsuda, Michiyo Hirano, Kazuko Saeki

**Affiliations:** ^1^Faculty of Health Sciences, Hokkaido University, Kita 12, Nishi 5, Kita-ku, Sapporo, Hokkaido 060-0812, Japan; ^2^Faculty of Health Sciences, Kansai University of International Studies, 1-18 Shijimicho Aoyama, Miki, Hyogo 673-0521, Japan

## Abstract

This study elucidated the skills of mothers who used to build positive interpersonal relationships with people they met through child-rearing. The research method was qualitative descriptive study. The participants were 24 mothers who had children between ages of one and four years. These participants were recruited at child-rearing salons and childcare centers located in various municipalities in Japan. The survey period was from September 2013 to July 2014. The mothers' interpersonal relationship-building skills were described by six categories: “ability to strive for new encounters,” “ability to try to interact socially with others,” “ability to choose compatible people,” “ability to continuously maintain good relationships,” “ability to take action suitable to a situation,” and “ability to build positive relationships with parents-in-law.” Cognitive aspects of assessing and understanding the interactions that occur during exchanges and behavioral aspects guided by these cognitions were identified within these skills. This study contributed to providing a framework to understand mothers' interpersonal relationship-building skills for public health nurses involved in child-rearing support.

## 1. Introduction

For many mothers, giving birth to a child means a clear break from the community to which they had previously belonged [1]. Kruse et al. [2] report that changes in mothers' social situations from the puerperal period to the child-rearing period are related to depression through lack of a sense of belonging. Similarly, Hagerty and Williams [3] report that lack of a sense of belonging, loneliness, and social support are powerful predictive factors of depressive symptoms; redeveloping positive interpersonal relationships is an imperative issue for postpartum mothers.

Immediately after giving birth to her child, a mother must build new relationships with people she meets through child-rearing and rebuild already-established relationships with friends and family as a mother. In modern Japan, however, there is a continuing trend toward nuclear families combined with a declining birthrate [4], couples' working patterns and lifestyles are diversifying [5], and economic disparity is becoming a problem. In current communities, the opportunities for child-rearing mothers to meet people with similar backgrounds in their neighborhoods are decreasing. Furthermore, the spread of individualism makes socializing even more difficult. According to Ogihara and Uchida [6], while European-American individualism is an active individualism, Japanese individualism has a tendency to preserve a steady sense of distance from other people or decide to end relationships. Uchida et al. [7] suggest that the spread of individualism in Japan is decreasing happiness from interpersonal relationships. In this societal background, mothers are placed in situations in which they must intentionally build child-rearing communities to obtain the social support necessary for their personal mental health and child-rearing. According to a 2013 report [8], the yearly number of cases at child counseling centers related to child abuse exceeded 73,000. Child-rearing isolated from regional communities is thought to be one of the factors.

Therefore, in previous research, the authors first clarified the people in the community mothers met through child-rearing [9]. From this study, we found that mothers' child-rearing communities must be intentionally made by mothers themselves and require a high degree of interpersonal relationship-building skills during opportunities for interaction. From exchanges among mothers in a child-rearing support program in England, Peters and Skirton [10] also report on the difficulty of finding close friends who can be mutually supportive during child-rearing.

This study focuses on the skills mothers use to build positive relationships with people. The known skills for building positive interpersonal relationships are emotional and social intelligence [11–13] and personal competence [13–15]. At the core of these interpersonal relationship skills are empathy [16, 17] and trust [18]. They are composed of skills including cognition and prosocial behavior [19, 20]. While referencing findings from these previous studies, this study clarified the skills mothers need to build positive relationships with the various people they meet during the initial stages of the child-rearing process.

## 2. Materials and Methods

To clarify the skills mothers need to build positive relationships with the various people they meet during the initial stages of the child-rearing process, we used the qualitative descriptive study.

### 2.1. Sample and Setting

The participants of this study were mothers who visited child-rearing support centers and salons. The sampling strategy was Quota sampling. Participant inclusion criterion was to be the mother of a child aged between 1 and 4 years. Working mothers were also included as study participants. It did not matter whether they had spouse or not, and the number of children and whether the mothers felt anxious regarding their children's health or not also did not matter. The total of participants was 24 mothers. When we began this study, we explained the study purpose and ethical considerations regarding the participants to the head of each facility orally and in writing. The study participants were chosen through staff mediation among the mothers who were attending the child-rearing salon that day. However, since social mothers tended to be selected through staff mediation, the author also recruited. At that time, five participants declined participation; two mothers refused the request and three mothers were forced to decline because of their intensely crying children. Ultimately, the author recruited four people and 20 people were recruited by staff at the child-rearing salon venue.

Since regional influences were expected to influence mothers' exchanges with others, we performed sampling from various local municipalities. When selecting the study area, we recruited 13 people at four child-rearing salon and childcare centers located in a city with population of around 1.9 million, three people from a municipality with population of around 90,000, two people from municipalities with population of around 20,000, and six people at three facilities located in a municipality with population of under 10,000.

### 2.2. Data Collection

The data collection method was individual interview and a semistructured interview. We created an interview guide for asking about the mothers' interaction experiences from the time they gave birth to their babies' present ages. A small room adjacent to the salon was prepared for conducting interviews and space was secured to maintain privacy. In addition, baby-sitting support staff was arranged so the mothers would be able to focus on the interview. Efforts were made to build an environment in which mothers could speak easily based on a relationship of trust between participant and researcher.

The data collection period was between September 2013 and July 2014. Interviews were recorded on an IC recorder and data was created with verbatim transcription. The length of the interviews ranged between 25 and 58 minutes depending on the participant, with an average of 36 minutes.

In this study, the scope of theory development was focused on mothers' interpersonal relationship-building skills and we aimed at theoretical saturation. We reached theoretical sufficiency at approximately 20 interviews. We then performed additional four interviews and finished by sampling all 24 interviews, ensuring the soundness of the generated theory. In addition, because most mothers' narratives were narratives of successful child-rearing, we examined reverse examples to investigate the generated theory's properties and dimensions. For the reverse examples, we conducted interviews with five experienced public health nurses engaged in child-rearing support and inquired about cases in which the mother could not sufficiently build relationships with others. The fieldwork consisted of 37 visits to 18 parenting salons for participant observation of mothers' behavior and interactions with staff and other mothers.

### 2.3. Data Analysis

We performed thematic analysis. The data were verbatim transcripts created using the sound recordings. We read the narrative data repeatedly in order to become familiar with latent mean in the mothers' story. Next, we extracted and coded data as initial codes focused on interpersonal relationship-building skills from the mothers' narratives on feelings and behaviors during episodes of interaction with people they met through child-rearing.

The initial codes were examined for similarities and integrated into secondary and final codes to create subcategories to explain specific concepts. To vividly describe the mothers' interactions with others, the subcategories attempted to represent the tone of the mothers' narratives to the extent possible. Furthermore, for in vivo codes such as “If our children are the same age, we can share the same problems”—a comment expressed by many of the mothers—the context of this comment's background and the experiences and shared awareness of participants who made this comment were carefully explored. In this way, subcategory characteristics were examined, while focused coding was conducted.

The characteristics of the subcategories such as “bravery, volition, sympathy, and assessing the situation” were carefully explored. Furthermore, we determined the main subcategory through exploring the relationships between subcategories and categories. These categories are the mothers' skills and are the core concepts that explain the theory in this study.

Researchers reviewed and checked codes and data as necessary in the process of categorization while conducting a posteriori analysis. During the theoretical coding process, we asked five mothers who participated in the interviews to do member checks [21] to ascertain the appropriateness of our data interpretation, and corrections were made if necessary. The participant observations conducted over multiple sessions were beneficial in verifying the appropriateness of the generated concepts and exploring new perspectives. In addition, throughout all parts of the research process, we obtained reviews by fellow researchers knowledgeable about public health nursing with a wealth of qualitative research experience.

### 2.4. Ethical Approval

The study's purpose and ethical considerations were explained orally and in writing to the mothers who agreed to cooperate; consent was received in writing. A small room adjacent to the salon was prepared for conducting interviews and space was secured to maintain privacy. In addition, baby-sitting support staff was arranged so the mothers would be able to focus on the interview. Efforts were made to build an environment in which mothers could speak easily based on a relationship of trust between participant and researcher.

This study was reviewed and approved by the research ethics committee of the Faculty of Health Sciences of Hokkaido University.

## 3. Results

### 3.1. Demographic Characteristics

The mothers' demographic characteristics are shown in Table 1. All mothers had spouses and no anxiety regarding their children's health. The impact of the mothers' backgrounds on the results was nullified in the end through the process of increasing the abstraction level in the categories. The participants included mothers who had one or more children; however, in the interviews, they mainly described episodes in raising their first child. Commonalities by categories were found, although there were differences in community exchanges by region of residence, dwelling history, and proximity to relatives.

### 3.2. Mothers' Skills Used to Build Positive Relationships with Others

The mothers' skills used to build positive relationships with others are shown in Table 2. Below we explain an overview of the categories. The subcategories are written in quotation marks.

#### 3.2.1. Ability to Strive for New Encounters

The representative subcategory of this category was “For my child's sake, I think I can gather courage and participate.” The mothers knew about the childcare support centers and child-rearing salons but were worried about getting along with the group of other mothers and found it difficult to participate by themselves. Therefore, even if they were unable to initiate conversations with other mothers, with the help of staff members they challenged themselves to have new encounters.

#### 3.2.2. Ability to Try to Interact Socially with Others

This category comprised subcategories related to cognitive aspects of sociability such as “I can generally build good relationships with anyone” and subcategories related to behavioral aspects such as “I try to be first one to say hello.” Within this category, there was a range of dimensions from positive to negative, such as* I would actually like to talk more with the other mothers, but I am not good at smoothly developing conversation.*

#### 3.2.3. Ability to Choose Compatible People

For the mothers, perceptions such as “It is difficult for me to maintain relationships if there are differences in our personalities and values” existed as premises for initiating interaction with other people. To verify this, they searched for commonalities in each other's lifestyles and other backgrounds. This extended to the act of physically approaching someone while determining her reliability.

#### 3.2.4. Ability to Continuously Maintain Good Relationships

Mothers estimated the benefits of establishing connections with another person, such as* I want to create relationships in which I can casually receive information when something happens to my child*. They are also consciously thinking of ways to sustain their social relationships, as explained by the code* If I remember the name of the other person's child, it is easy for me to say hello when I see them in the neighborhood*. The subcategory “I want to be in mutually caring relationships with friends” was a concern for maintaining good relationships with each other.

#### 3.2.5. Ability to Take Action Suitable to a Situation

Mothers were choosing conversation topics while calculating the closeness/intimacy with the other person. At the same time, they were determining the fine sense of distance that was neither too close nor too distant and were preserving that distance as they developed interactions. Although troubles among children such as little fights over toys were common, other mothers' attention to this trouble was an important element in building positive relationships among mothers.

#### 3.2.6. Ability to Build Positive Relationships with Parents-in-Law

In Japan, relationship-building between the wife and the husband's parents is especially important. By having children, mothers felt an even stronger connection to their husbands' families. This category describes the skills mothers utilize in this setting.

Mothers sensed that their in-laws wanted to see their grandchild regularly so they intentionally asked for their support. However, they sometimes held back, concerned about the burden on their in-laws; while calculating the subtleties, they sought a positive relationship and sense of distance for both sides. They handled conflicts in child-rearing methods by being respectful of their in-laws' thinking while taking care not to wound their pride.

## 4. Discussion

The skills mothers used to build positive relationships with people they met during child-rearing were explained by six categories. These categories are abilities and skills that are required in every step of interpersonal relationship. “Ability to strive for new encounters,” “ability to try to interact socially with others,” and “ability to choose compatible people” are required at the first step of making friends in opportunities such as child-rearing support centers.

The next step in developing relationships is keeping and developing the connection. “Ability to continuously maintain good relationships” and “ability to take action suitable to a situation” are important skills in this step. According to Kooker et al. [22], self-management is an important interpersonal competency. Self-management includes controlling self-destructive or impulsive emotions, striving for sincerity and honor in contact with others, and a goal-oriented mindset. We understood mothers also required these competencies.

The relationship with parents-in-law was narrated by many mothers as an important experience in their child-rearing. This phenomenon may be indigenous in Japanese family culture. In Japan, it is often said that getting married means marrying one's husband's family. Thus, most mothers are required to build a good relationship with their parents-in-law. In fact, their parents-in-law provide concrete support such as financial aid. Thus, “ability to build positive relationships with parents-in-law” may be an important skill in their child-rearing.

In addition, Barone et al. [23] describe social network orientation as individual beliefs or attitudes that people have when seeking new networks for gaining support and estimating and determining how helpful accessing those networks would be. We also could confirm social network orientation in this study outcome. As described above, this study's results contributed to providing a more concrete understanding and were compatible with existing theories.

For today's mothers, the skills elucidated in this study are necessary skills for being able to independently raise children so that they can smoothly accept support from others and create better personal child-rearing support. In addition, the categories confirmed cognitive and behavioral skills related to Japan-specific values cherishing peace and harmony and maintaining a delicate sense of distance with others.

The reality of weakening child-rearing support in local communities and support activities for rebuilding that support have become important issues not only in Japan but also in other countries [10]. The interpersonal relationship skills discussed in this study are both teachable and learnable [24, 25]. Rather than attributing the responsibility of child-rearing to mothers individually, child-rearing support specialists are calling for support that helps develop these skills while accumulating successful relationship-building skills one at a time through child-rearing. For public health nurses involved in child-rearing support, this study's results contribute to the framework of understanding mothers' interpersonal relationship-building skills.

The Japanese government began a new policy in 2014, including child-rearing support center projects. Some home visiting services are also operated under this new policy [26]. Public health nurses working for municipalities are key persons in these projects. This outcome is useful to find mothers who need support and how public health nurses can help those mothers. This is also important knowledge for developing support systems.

Lastly, we describe study limitations. This study's participants had no anxiety about their children's health conditions and were healthy and relatively sociable mothers who were able to attend child-rearing support centers and salons of their own volition. Therefore, more specific data may be extracted from cases with mothers raising children who are ill or disabled, mothers who are ill or disabled, and single mothers. To build relationships with others, it is also necessary to be motivated to want to connect with others. This paper was unable to address these points. In addition, we could not explore the relationship between each element of mothers' interpersonal abilities, priorities, and so forth. We would like to address these challenges in the future.

## 5. Conclusions

This study clarified the skills mothers used to build positive relations with people they met during child-rearing. The mothers' skills elucidated in this study are supported by previous studies and provided a more concrete understanding of these theories. In addition, for mothers, the skills elucidated in this study are necessary skills for independently raising children so that they can smoothly accept support from others and create a better personal child-rearing support team in communities that have recently been experiencing weakening interpersonal connections. Moreover, this study confirmed cognitive and behavioral skills that relate to Japan-specific values cherishing peace and harmony.

## Figures and Tables

**Table 1:** Mothers' demographic characteristics.

Variable	Values	*n*
Age	25–29	1
	30–34	6
	35–39	13
	40–44	4
Employment		5
Education	High school	11
	Vocational/junior college	3
	University	10
Number of children	One	14
	Two	7
	Three or more	3
Residence	Single-family house	9
	Apartment house	15
Area^*∗*^	Urban	13
	Rural	11
Dwelling history (year)	<1	3
	1–4	11
	>5	10
Frequency meeting their parents	Once a week	1
	Once a month	16
	Twice or three times per year	4
	Less than once a year	3

^*∗*^Urban: population more than a million.

**Table 2:** Mothers' skills to build positive relationships with others.

Categories	Subcategories	Typical codes
Ability to strive for new encounters	It is difficult to participate in something by myself, even if I already know about it.	(i) I knew about the facility, but I just couldn't gather the courage. (ii) I feel uneasy entering an already-established group.
	With play plans and staff mediation, I can participate comfortably.	(i) Without any mediation, it would be impossible to make friends. (ii) It became easier for me to participate because the staff gave their kind attention.
	For my child's sake, I think I can gather courage and participate.	(i) I am tense going to places where I don't know anybody, but if my child says he/she wants to go, I'll go. (ii) Going somewhere new is extremely scary for me, but for my child's sake, I was brave.

Ability to try to interact socially with others	I try to be first one to say hello.	(i) When I meet someone I know, I try to be the one to initiate a greeting. (ii) I am the one to begin talking about my child's growth and development.
	I can generally build good relationships with anyone.	(i) I think it is a loss to be shy. (ii) I find having conversations with others enjoyable.

Ability to choose compatible people	It is difficult for me to maintain a relationship if there are differences in personalities and values.	(i) I cannot socialize unless the other person behaves with decent common sense. (ii) While talking to another mother, I can tell if we are compatible.
	I look for commonalities such as shared topics and mutual experiences.	(i) It may be easier to become friends if we have similar lifestyles or family composition. (ii) If our children are the same age, we can share the same problems.
	I discern how trustworthy a person is.	(i) I am wary of people who ask about my husband's profession the first time we meet.
	I sense if someone has an approachable atmosphere.	(i) If the other person doesn't seem to want to interact, it is difficult for me to strike up conversation.

Ability to continuously maintain good relationships	I estimate the benefits of having connections.	(i) I want to create relationships in which I can casually receive information when something happens to my child. (ii) I build relationships now anticipating that they will continue in the future.
	I want to continue good social relationships.	(i) If I remember the name of the other person's child, it is easy for me to say hello when I see them in the neighborhood. (ii) Over time, I would like to connect with all sorts of people.
	I want to be in mutually caring relationships with friends.	(i) When I see someone who has no one to rely on, I want to help that person somehow. (ii) Because we both understand each other's busy situations, I don't mind if email replies are late.

Ability to take action suitable to a situation	I choose conversation topics depending on the other person.	(i) I have different topics for friends without children. (ii) It is tiresome to be told about child-rearing ways from the old days, so I do not really bring up the topic of child-rearing when speaking with old women in the neighborhood. (iii) I pay attention when choosing my words so as not to make the other person uncomfortable.
	I will approach in harmony with those around me.	(i) In regards to doing anything, I think it is better to be a little reserved if possible. (ii) Balance is important because if you put up too strong of a wall of defense, it is impossible to become close with someone.
	Concern regarding trouble among children.	(i) Little fights over things are to be expected among children, so I tell the other mothers not to worry about them. (ii) I think it is important for parents to watch their children closely and be able to say “sorry” if something happens.

Ability to build positive relationships with parents-in-law	I value the time I spend with my parents.	(i) I hold events such as the 100th day after birth and birthday celebrations together with my parents. (ii) I make my parents happy by giving them photographs of my child and things that he/she made. (iii) I sense that my in-laws also want to look after their grandchild.
	I indulge in their kindness while remaining reserved.	(i) My in-laws will feel uncomfortable if I am too reserved, so I say thank you and indulge in their kindness. (ii) I think we should look after our child's affairs as a couple as much as possible.
	I try to show deference to my parents-in-law.	(i) When my in-laws and I have a difference of opinion, I try to present it as information that I heard from someone else so as not to offend them. (ii) Each time I have a request, it would be easier to ask my own parents, but I withhold it and show deference to my husband's parents.

## References

[1] Alstveit M., Severinsson E., Karlsen B. (2010). Obtaining confirmation through social relationships: Norwegian first-time mothers' experiences while on maternity leave. *Nursing and Health Sciences*.

[2] Kruse J. A., Williams R. A., Seng J. S. (2014). Considering a relational model for depression in women with postpartum depression. *International Journal of Childbirth*.

[3] Hagerty B. M., Williams R. A. (1999). The effects of sense of belonging, social support, conflict, and loneliness on depression. *Nursing Research*.

[4] World Health Organization, World Health Organization (2010). Demographic and socioeconomic statistics. *World Health Statistics 2010*.

[5] Cabinet Office ‘Males’ work and life in transition. http://www.gender.go.jp/english_contents/about_danjo/whitepaper/pdf/ewp2014.pdf.

[6] Ogihara Y., Uchida Y. (2014). Does individualism bring happiness? Negative effects of individualism on interpersonal relationships and happiness. *Frontiers in Psychology*.

[7] Uchida Y., Kitayama S., Mesquita B., Reyes J. A. S., Morling B. (2008). Is perceived emotional support beneficial? Well-being and health in independent and interdependent cultures. *Personality and Social Psychology Bulletin*.

[8] http://www.mhlw.go.jp/toukei/list/38-1a.html.

[9] Honda H., Matsuda N., Hirano M., Saeki K. (2014). Significance of social support in mothers' communities created through the child-rearing process. *Bulletin of Health Sciences Kobe*.

[10] Peters J., Skirton H. (2013). Social support within a mother and child group: an ethnographic study situated in the UK. *Nursing & Health Sciences*.

[11] Amdurer E., Boyatzis R. E., Saatcioglu A., Smith M. L., Taylor S. N. (2014). Long term impact of emotional, social and cognitive intelligence competencies and GMAT on career and life satisfaction and career success. *Frontiers in Psychology*.

[12] Kaukiainen A., Björkqvist K., Lagerspetz K. (1999). The relationships between social intelligence, empathy, and three types of aggression. *Aggressive Behavior*.

[13] Mayer J. D., Caruso D. R., Panter A. T., Salovey P. (2012). The growing significance of hot intelligences. *American Psychologist*.

[14] Griffin K. W., Scheier L. M., Botvin G. J., Diaz T. (2001). Protective role of personal competence skills in adolescent substance use: psychological well-being as a mediating factor. *Psychology of Addictive Behaviors*.

[15] Reason R. D., Terenzini P. T., Domingo R. J. (2007). Developing social and personal competence in the first year of college. *The Review of Higher Education*.

[16] Barrett-Lennard G. T. (1981). The empathy cycle: refinement of a nuclear concept. *Journal of Counseling Psychology*.

[17] Morse J. M., Anderson G., Bottorff J. L. (1992). Exploring empathy: a conceptual fit for nursing practice?. *Image*.

[18] Reynolds W. J., Scott B. (1999). Empathy: a crucial component of the helping relationship. *Journal of Psychiatric and Mental Health Nursing*.

[19] Cantor N., Kihlstrom J., Wyer R. S., Srull T. K. (1989). Social intelligence and cognitive assessments of personality. *Advances in Social Cognition. Volume II: Social Intelligence and Cognitive Assessments of Personality*.

[20] Ford M. E., Tisak M. S. (1983). A further search for social intelligence. *Journal of Educational Psychology*.

[21] Holloway I., Wheeler S. (2002). *Qualitative Research in Nursing and Healthcare*.

[22] Kooker B. M., Shoultz J., Codier E. E. (2007). Identifying emotional intelligence in professional nursing practice. *Journal of Professional Nursing*.

[23] Barone C., Iscoe E., Trickett E. J., Schmid K. D. (1998). An ecologically differentiated, multifactor model of adolescent network orientation. *American Journal of Community Psychology*.

[24] Benson G., Ploeg J., Brown B. (2010). A cross-sectional study of emotional intelligence in baccalaureate nursing students. *Nurse Education Today*.

[25] Ozcan C. T., Oflaz F., Sutcu Cicek H. (2010). Empathy: the effects of undergraduate nursing education in Turkey. *International Nursing Review*.

[26] Cabinet Office http://www8.cao.go.jp/shoushi/shinseido/event/publicity/pdf/naruhodo_book_2609/eng/print.pdf.

